# Axiological reflection for nursing ethics education: The missing link in understanding value conflicts

**DOI:** 10.1177/09697330241295369

**Published:** 2024-10-24

**Authors:** Johanna Elise Groothuizen

**Affiliations:** 4616 King’s College London

**Keywords:** Axiology, moral values, personal values, practical reasoning, nursing ethics education, hidden curriculum

## Abstract

Texts from various areas of the world highlight the importance of moral values like compassion and integrity in healthcare. Such values are held in high esteem by healthcare organisations and are actively ‘taught’ within nursing ethics education to ensure their presence within the future workforce. With such an emphasis, it is easy to overlook that moral values are not the only values that people, including nurses, hold. *Other* personal values – which may or may not conflict with moral values – are simultaneously present within individuals. Therefore, moral behaviour cannot be predicted solely by the presence/absence of certain moral values. Instead, it depends on how these integrate into an individual’s broader values system.

Using Schwartz’s axiological Theory of Basic Human Values as a framework, I argue that moral values are but one part of an individual’s greater personal values spectrum, which also includes, for instance, hedonism, achievement, and power. Within this spectrum, values are ordered hierarchically, influencing behaviour based on relative priority. When a conflict arises between moral and other personal values, the prioritisation of moral values is a requirement for moral behaviour.

I discuss how socialisation in suboptimal clinical practice environments can cause moral values to be deprioritised and argue that the development of practical reasoning skills is paramount to learning to balance one’s values and guide decision-making. I advocate for the integration of (meta-)axiological reflection – characterised by introspection and aimed at developing a deeper understanding of one’s personal values spectrum – within nursing ethics education. This involves exploring the origin, meaning, and perceived relative importance of one’s different personal values. By incorporating specific reflective exercises, students can increase self-awareness/insight and enhance their ability to recognise situations where conflicts between their moral values and other personal values may occur, which is likely to benefit moral decision-making in clinical practice.

## Introduction 

In the context of healthcare provision, the importance of ‘values’ is widely recognised. This is evidenced in the large number of texts from across the globe, for example,^1–4^ discussing values that should underpin care and patient interactions. Given that values can have different interpretations across contexts^5^ and considering the impact of cross-cultural diversity, specific emphases may vary. However, there appears to be relative international consensus around compassion, respect, integrity, justice, and commitment to excellence as crucial healthcare values.^1–4,6^

The discourse regarding values has prompted national and international policymakers and organisations to develop numerous organisational value lists and statements that they expect care personnel to adhere to, extending to the nursing profession. At a macro-level, there are values statements from international bodies such as the International Council of Nurses.^7^ In the United Kingdom, there are statements applying to all who work within the National Health Service (NHS), such as the NHS Constitution values^8^ and the ‘six Cs’ (i.e. Care, Compassion, Courage, Communication, Commitment, and Competence),^9^ as well as additional nursing-specific lists outlined in the ‘Nursing and Midwifery Council Code’^10^ and the Royal College of Nursing’s ‘Principles of Nursing Practice’.^11^ This is merely the beginning, as individual Trusts, hospitals, and surgeries develop their own, local values statements.^12^

At any level, values statements applicable to the nursing profession specify ‘correct’ ways of caring for, and interacting with, patients, underscoring an inherent ethical focus. In line with the above, they tend to include words like *patient-centeredness*, *compassion*, *respect*, *dignity*, and *quality of care*.^13^ These elements can be linked to key concepts within contemporary virtue ethics-based healthcare scholarship, such as benevolence, respectfulness, and trustworthiness.^14^ Virtue ethics is the branch of normative ethics concerned with moral character, as opposed to duty (deontology) or the consequences of actions (consequentialism).^15^ Arguably, the alignment of nursing values statements with virtue ethics especially suggests a focal point – among organisational leaders – on the *character* of nurses, referring to their goodness as *people*, rather than the moral rightness of actions.

Concerns have been raised, however, around the usefulness of organisational values statements. Gallagher^16^ argues that ‘checklists’ of values may ‘deaden our hearts rather than stimulate our moral imagination’, reducing the multifaceted reality of caring to a simple list of bullet points where a more speculative exploration^17^ would be warranted. Puig de la Bellacasa^17^ pleads in favour of a more nuanced approach to valuing, and embracing a more complex understanding of, care. Regarding ethics, we should, in her view, aim to engage deeply with the messy realities of caring in an interconnected world. This includes various associated complexities, uncertainties, and ambiguities.^17^ Moving away from simplistic notions and adopting a more comprehensive viewpoint on values, we need to recognise that moral values deemed desirable for the nursing profession (and proudly proclaimed in organisational statements) are not the only values that nurses hold; as human beings, they inherently hold other personal values as well. These may or may not conflict with moral values and/or interfere with the extent to which moral values predict behaviour.

In this philosophical paper – informed by social psychological theory and research – I discuss how focussing on moral values without acknowledging other personal values is problematic, leading to gaps and oversights within nursing ethics education. I provide recommendations in relation to the inclusion of reflection around wider values, in order to establish a more well-rounded curriculum.

### The nature of values and axiology

As the nursing profession tries to negotiate the overload of moral values statements that is being imposed,^16^ it is easy to forget that values are not inherently moral at all. A value – as defined by Rokeach^18^[p.5] – is simply ‘an enduring belief that a specific mode of conduct or end state of existence is personally or socially preferable to an opposite or converse [one]’. Within this notion, people can hold values as *‘just’ preferences* and values as *principles*^19^; the former essentially being ‘attitudes’ (e.g. favouring particular environments and situations), whereas the latter refers to more deeply ingrained general beliefs regarding how one ought to behave.

Values as principles are our personal values, reflective of what we find important; they strongly link to feelings and motivational goals, which serve as standards for the decision-making-processes that ultimately underpin our actions.^20^ As, when it comes to nursing, this link between values and behaviours is our primary interest, further references to ‘values’ in this paper will, along the lines of Parks and Guay’s 2009 article,^19^ implicitly refer to such personal values. I acknowledge the existence of different stances as to whether values are objective or subjective and individual or societal. While this debate is largely outside the scope of this paper, it is, in this respect, important to point towards the co-existence of, and distinction between, personal values and *‘norms’*. Norms are *societal* values and standards, present within certain groups.^20^ Personal values affect whether norms are accepted or rejected.^20^

Axiology is the discipline that studies values in general, rather than moral values in particular. Axiology acknowledges the plurality and heterogeneity of values.^21^ Regarding personal values specifically, many axiological theories exist. The most developed and widely used^19^ theory is Schwartz’s ‘Theory of Basic Human Values’.^20^ I use this framework here to illustrate how moral values are but one part of a wider spectrum of personal values.^22^ Building on historical axiological scholarship, the theory places different personal values in a circular arrangement, representing a continuum of underpinning motivational goals, with values closer to each other representing more similar goals.^20^ A distinction is made between growth versus self-protection/anxiety-avoidance, and individually versus socially focused values.^23^

Schwartz does not explicitly state that these are ‘ultimate’ values but argues that the theory’s universality lies in the fact that all these values stem from the ultimate goal to successfully cope with the requirements of human existence: people’s needs as biological organisms, and their needs for coordinated social interaction, survival, and welfare. Addressing these aspects, the theory is regarded as comprehensive, with all possible human motivational goals mapping onto one or more of these 19 values. It has been tested across a wide range of countries in different parts of the world.^20^

Table 1 includes values that closely align with morality, such as Benevolence and Universalism, as well as many co-existing values *without* a moral focus, such as hedonism, achievement, or power.^20,23^ Beliefs regarding how we ‘ought to’ behave do not necessarily refer to moral correctness: Someone who is preoccupied with enjoying themselves may believe that they ‘ought to’ do what is most enjoyable and avoid engaging in less pleasurable activities, whereas someone focused on status may, for instance, be motivated to act in a way that increases their chances of ascending the leadership ranks. Within individuals, values exist in a hierarchical structure and are ordered according to the relative importance placed on them.^20^ As long as values higher and lower down in one’s personal hierarchy are compatible with each other, individuals may act in accordance with either or both. However, when conflicts or challenges between values arise, values higher up in the hierarchy are more likely to predict behaviour.^20^

**Table 1.** 19 basic human values.^23^

Values (as defined by Schwartz)	Referring to
Self-direction – Thought	Creativity, curiosity, and interest
Self-direction – Action	Independence and self-reliance
Stimulation	Excitement, novelty, and challenge
Hedonism	Pleasure and enjoyment
Achievement	Being successful by the standards of others
Power – Dominance	Being in charge
Power – Resources	Wealth
Face	Maintaining one’s public image
Security – Personal	Staying healthy, safe, and avoiding danger/illness
Security – Societal	Social order and stability
Tradition	Cultural and religious traditions
Conformity – Rules	Conforming to rules and formal obligations
Conformity – Interpersonal	Avoiding causing upset to others
Humility	Being modest and not being boastful
Universalism – Nature	Protecting the environment
Universalism – Concern	Equality and social justice
Universalism – Tolerance	Broad-mindedness and acceptance
Benevolence – Care	Being helpful and promoting the welfare of others
Benevolence – Dependability	Being reliable and trustworthy

While axiological frameworks such as Schwartz’s include moral values as one component of a greater values spectrum, moral philosophy generally does not encompass the wider field of axiology.^22^ Despite moral values being a subset of values, many ethicists tend to research them in relative isolation, with little acknowledgement of the wider values context in which they exist.^22^ One can argue that this is like trying to assemble a jigsaw puzzle where some of the pieces are missing, making it impossible to get sight of the complete picture.

### Presence versus prioritisation of values

In line with the NHS’s focus on values, UK nursing education programmes assess students for certain values before offering them a place.^12^ Subsequently, nursing ethics education actively teaches values statements,^13^ as part of a ‘competency-based’ curriculum.^24^ There appears to be a strong focus on ensuring that certain values are *present* in future nurses.

This is also evident in the aims and objectives of many nursing education studies. A review of literature reveals an abundance of nursing ethics education research focused on determining – through surveys, interviews, and other self-reported methods – the respective *presence* or *absence* of moral values in students.^13^ This dichotomous view, which does not acknowledge the simultaneous presence and potential influence of other personal values when it comes to predicting behaviour, is concerningly reductive. I argue that ethics education within nursing curricula falls victim to the same ‘missing puzzle pieces’ problem – or as Kupperman describes it, ‘partial blindness’ – that tends to characterise moral philosophy in general.^22^ Considering that values exist within a hierarchy,^20^ a requirement for moral motivation and subsequent moral behaviour is the prioritisation of moral values over other personal values when a conflict or clash between these different types of values arises.^25^ The mere *presence* of moral values within a person is therefore of little relevance if these are not prioritised over other values.

### Moral courage or moral motivation?

Whilst I speak of value prioritisation in an individual, personal sense here, it should be noted that personal values are not impervious to social and cultural factors. Although values are relatively stable across situations, the fact that they are acquired through socialisation means that interaction with the environment can cause changes to individuals’ values systems. When nursing students enter the clinical practice environment, a new sense of occupational identity is acquired, meaning that old roles and self-conceptions are abandoned in favour of new ones, and that norms, attitudes, and – indeed – values typical of qualified practitioners are internalised.^26,27^ Environments which accentuate particular values can ultimately cause individuals’ values to shift in this direction.^28^ In addition to the formal educational curriculum, this implicit, practice-based learning is the ‘informal’ or ‘hidden’ curriculum of nursing.

Absorbing the hidden curriculum is inevitable and, whilst this can be valuable in helping individuals adapt to their roles within healthcare institutions, it can also be problematic. According to MacIntyre,^29^ there may be a ‘double character’ to institutionalised relationships. Such relationships are, on one hand, conducive to achieving common goods (i.e. to benefit patients) but, on the other hand, characterised by established hierarchies that bring potential for domination and deprivation in practice situations, privileging the interests of those in power, whilst marginalising others. The reality of clinical practice environments does not always align with the lists of values adorning the hospital walls, and can be characterised by task- rather than person-focused care^30,31^ and unprofessional behaviours.^32^ In this context, the hidden curriculum can refer to a negative socialisation climate,^33^ where repeated exposure to suboptimal norms and practice inadvertently teaches students to deprioritise moral values.

Negative effects of clinical practice environments on nurses’ moral values have been demonstrated in multiple studies. Maben,^31^ for instance, showed that the wish to fit into the workplace and be part of the team has the potential to compromise and override previously existing care provision ideals. My own research,^13^ which utilised a Situational Judgement Test featuring written scenarios grounded in Schwartz’s theory, examined nursing students’ responses to clinical practice situations pitting moral values against conflicting ones. Findings suggested that moral values can be deprioritised in environments involving a performance element: students showed a willingness to accept, or be complicit in, poor practice initiated by more senior staff, as they strived for clinical placement success. Due to the culture in clinical practice environments, they were concerned that speaking out or advocating for patients would compromise this.

When it comes to these less-than-ideal clinical practice situations, where moral aspects become overshadowed by pressures to perform or comply, the need for moral courage is often raised. Moral courage is described as ‘a person’s capacity to overcome fear and *stand up for their core values* and moral obligations’.^34^ Values in this context, again, appear to be understood as inherently moral; there is an implication here that we are, ultimately, morally motivated, and that we just need to be brave enough to act accordingly. Stating that we need to ‘stand up for our values’ in a difficult situation, suggests that the experience of difficulty exists *outside of our values*. When we approach this through an axiological lens, however, the segregation between (moral) values as internal and constraints as external – also seen in the vast body of literature covering the topic of moral distress^35^ – seems incorrect. I propose here that our perception of, and reaction to, ‘external’ constraints is, in fact, very much intertwined with our personal values hierarchy and prioritisation: a preoccupation with ‘fitting into the team’ could imply that values in relation to interpersonal conformity^20,23^ have become prioritised over moral values. Not wanting to speak out in the context of poor practice due to a fear of not passing a placement may be indicative of a prioritisation of self-enhancement/achievement values^20,23^ over moral considerations. This is concerning in a field like nursing, where morally driven decision-making is paramount. Branco^36^ argues that moral values underpin the formation of people’s ethics and morality. Considering this, the deprioritisation of moral values at such an early stage in students’ careers could hamper their moral development as nurses, potentially with long-lasting effects.

Contributing to negative socialisation – where nurses’ values hierarchies may gradually shift, to the detriment of moral values – may be a sense of cognitive dissonance experienced upon recognising that the reality of clinical practice environments does not always align with moral values. As cognitive dissonance is unpleasant, nurses can put up a ‘smoke screen’ of justifications, trivialisations, denial, or shifts in attention to rid themselves of it.^37^ Research also supports the notion that witnessing moral violations by others inhibits people’s personal moral motivation, bringing to the forefront goals pertaining to hedonism and personal gain.^38^ In the absence of moral motivation,^25^ moral courage is largely irrelevant.

### Inclusion of axiology within nursing ethics education

In line with Kupperman’s reflections,^22^ axiology is not commonly included within nursing ethics education. In my search for literature regarding personal values in nurse education, I found some questionnaire-based observational studies examining the presence of these values in students^39–41^ and a single suggestion^39^ that embedding these in curricula might be advantageous – but no evidence of any instances where this had been implemented. The closest approximation is, perhaps, the teaching of ‘vices’, as the inverse or mirror-image of virtues^42^ within virtue ethics education. However, much like the ‘presence-absence’ view in relation to moral values,^13^ this approach lacks reference to a wider context of personal values. Where educational discussions regarding values conflicts *do* take place, these often focus on ‘moral dilemmas’,^43^ referring to situations where two or more desirable moral values clash with one *another (personal moral values versus personal moral values)*. This is, for instance, the case when a nurse has to choose between respecting patient autonomy (honouring a patient’s decision) and beneficence (acting in the patient’s best interest), or between truthfulness (e.g. disclosing an unfavourable prognosis in full detail) and non-maleficence (avoiding causing (psychological) harm to the patient). A different type of dilemma sometimes covered in nursing ethics education is one where, in a certain context, individuals’ personal beliefs about what is best for the patient, or morally correct, conflict with prevailing societal or institutional moral norms *(personal moral values versus institutional moral values)*. Regarding abortion or euthanasia, for instance, differing views on what is morally right have led to debate and conscientious objection. Whilst learning to negotiate dilemmas involving conflicting moral values is crucial for becoming a nurse, it is determinately different to resolving conflicts between personal moral values and other, *non-moral* personal values (see Table 1).

Since its first conceptualisation by Kramer^44^ in 1974, much research has focused on the ‘reality shock’ that novice nurses face when moving from the educational setting into the clinical practice environment. This arises from the discrepancy between the holistic, patient-centred approach emphasised in education and the realities of practice, which are shaped by the pressures and power hierarchies previously mentioned. The experience of the reality shock can cause moral values and ideals to be abandoned or changed to adapt to the work situation.^30,45^ Insecurity regarding one’s professional role may exacerbate this process.^30^ I argue that teaching students (lists of) moral values without explicitly addressing the broader axiological context contributes to an unrealistic, one-dimensional view of the profession, further widening the theory-practice gap.^46^ As such, it may increase the magnitude of the reality shock and cognitive dissonance that students experience upon entering practice, and their perceived need to put up a ‘smoke screen’^37^ as a coping mechanism.

The fact that students will inevitably hold personal values that conflict with the moral values deemed desirable for the nursing profession needs to be explicitly acknowledged within nursing ethics education. In the next section, I discuss how students may acquire awareness and understanding of their values through reflective exercises.

### Axiological reflection and practical reasoning

To cope with the complexity of ethical decision-making, many have emphasised the importance of practical reasoning capability, which can inform and guide our pursuit of values by helping us evaluate options.^47^ MacIntyre^29^ discusses the importance of practical reasoning to inform behavioural judgements (‘why should I do this rather than that?’): Practical reasoners know how to detach themselves from their immediate desires, recognise where practical judgements are warranted, and are able to make such judgements appropriately.^29^ Sellman^48^ refers to ‘professional phronesis’ within the nursing context, arguing that this is necessary to discern what is right in the context of competing demands from different forces. The key here is awareness. Perron^49^ talks about the importance of Foucault’s ‘care of the self’ within nursing, which she relates to the dispositions of disobedience (an act of reason and agency) and parrhesia (speaking freely and fearlessly). She argues that ‘care of the self’ refers to a committed relationship that is developed with oneself through embodied attitudes and practices. This implies a questioning and challenging mindset directed at oneself in the first instance, examining one’s knowledge, but also the processes through which this knowledge came to exist, and the reasons why certain beliefs are maintained while others are not.^49^ Discussion exists as to whether nursing education should, to a greater extent, include meta-ethics, which involves reflections on the nature of ‘good’^50^ I take this a step further and argue that we should teach a meta-*axiology*, with nursing students being encouraged to reflect on the wide range of values – moral and other – that they hold; their origin and meaning, how these different values relate to one another, and, following on from this, where, how, and why conflicts might occur. Reflection is a foundational element in the development of practical reasoning skills^51^ and this can be informed by the deep introspection inherent to ‘care of the self’.

Nursing ethics education should provide space for open discussions around the potential impact of all types of values on decision-making processes in the clinical practice environment. Reflection within nurse education already routinely takes place in the form of, for instance, journal and diary entries, facilitated group discussions,^52^ scenario-based exercises, and Schwartz rounds.^53^ Adding axiological and meta-axiological components to such reflective exercises shifts the focus from a traditional epistemology to one in the service of ontology.^54^ This means that, rather than teaching students (lists of) moral values per se, we emphasise reflectivity, and the use of reflective exercises and skills to deepen one’s self-understanding. Frameworks like Schwartz’s^20,23^ might be used to guide students’ initial reflection on their own moral and other values, after which they can engage in further exploration as to where their values come from, what they mean, and how they are prioritised. Once this understanding is developed, scenario-based exercises could be used, with students identifying the salience/instantiation of their moral and other values across situations. This will help them gain a deeper insight into values conflicts that may occur which, in turn, is likely to benefit decision-making processes around potential courses of action.

## Conclusion

In this paper, I have argued that teaching moral values in isolation from other personal values may lead students to develop incomplete and inaccurate expectations of the nursing profession and the complexities of moral decision-making. Since the ‘reality shock’^44^ arises from unrealistic expectations, this approach may exacerbate the shock and potentially contribute to the eventual abandonment of moral values. To better prepare nursing students for practice, it could be helpful to start axiological reflection, as suggested above, prior to students’ first clinical placement, making use of relevant case studies and examples. Once students have engaged in practice learning, further scenarios for reflection could be drawn from their personal experiences within the clinical environment. The development and implementation of a curriculum that incorporates broader axiology and encourages axiological reflection offers a promising area for action research. This approach could yield valuable insights into the impact of increased values awareness among students and inform the refinement of educational strategies.

Gaining a deep, explicit understanding of (the nature and origin of) their own values, and how these are ordered within a personal hierarchy, may result in an enhanced ability to recognise situations where conflicts between moral values and other personal values may occur, and – consequently – identify where additional effort may be required to sustain moral behaviour. Let us aim to educate balanced, introspective future nurses, equipped with all the tools required to assemble their personal values puzzle in its entirety.
